# ApoE: The Non-Protagonist Actor in Neurological Diseases

**DOI:** 10.3390/genes15111397

**Published:** 2024-10-30

**Authors:** Lorenzo Grimaldi, Eleonora Bovi, Rita Formisano, Giulia Sancesario

**Affiliations:** 1 Clinical Neurochemistry Unit and Biobank, IRCCS Santa Lucia Foundation, Via Ardeatina, 306/354, 00179 Rome, Italy; 2 European Center for Brain Research, Via del Fosso del Fiorano, 00143 Rome, Italy; 3 Parkinson’s Disease Unit, University Hospital of Rome “Tor Vergata”, Viale Oxford 81, 00133 Rome, Italy; 4 Post-Coma Unit and Neurorehabilitation, IRCCS Santa Lucia Foundation, Via Ardeatina, 306/354, 00179 Rome, Italy

**Keywords:** apolipoprotein E, neurodegenerative diseases, Alzheimer’s disease, Parkinson’s disease, neuroinflammation, stroke, dementia, brain injury

## Abstract

Background: Apolipoprotein E (*APOE* = gene, ApoE = protein) is a glycoprotein involved in the biological process of lipid transportation and metabolism, contributing to lipid homeostasis. APOE has been extensively studied for its correlation with neurodegenerative diseases, in particular Alzheimer’s disease (AD), where the possession of the epsilon 4 (E4) allele is established as a risk factor for developing AD in non-familiar sporadic forms. Recently, evidence suggests a broad involvement of E4 also in other neurological conditions, where it has been shown to be a predictive marker for worse clinical outcomes in Parkinson’s disease (PD), brain trauma, and disturbances of consciousness. The mechanisms underlying these associations are complex and involve amyloid-β (Aβ) peptide accumulation and neuroinflammation, although many others have yet to be identified. Objectives: The aim of this review is to overview the current knowledge on ApoE as a non-protagonist actor in processes underlying neurodegenerative diseases and its clinical significance in AD, PD, acquired brain trauma, and Disorders of Consciousness (DoC). Ethical implications of genetic testing for APOE variants and information disclosure will also be briefly discussed.

## 1. Introduction

Neurological diseases (NDs) comprehend an ample variety of conditions affecting the central nervous system (CNS) of varying etiologies, e.g., developmental disorders, traumatic or vascular brain damage, tumors, infectious or autoimmune and neurodegenerative diseases of both early and late onset, with high economic and social costs. Although the pathogenic processes may be different, generally, NDs are characterized by neuronal death and atrophy, inflammation, oxidative stress, and, in some cases, the formation of proteopathic aggregates formed by insoluble neurotoxic misfolded proteins, which may aggregate into pathological toxic species which affect plasticity and synaptic functions. The most common neurodegenerative pathologies, such as Alzheimer’s (AD), Parkinson’s disease (PD), and Huntington’s disease (HD), are chronic progressive multifactorial diseases, caused by multiple genetic and environmental factors, currently affecting more than three billion patients worldwide, with a prevalence expected to grow rapidly as the average age of the world’s population increases [1,2]. Given modern large-scale sequencing technologies and sequencing studies on large populations, some genetic risk factors for neurological diseases have been identified, which may be associated with an increased incidence but also with more severe clinical phenotypes, and rapidly progressive malignancies. The apolipoprotein E (*APOE*) gene is now recognized as the most influential genetic factor in sporadic late-onset Alzheimer’s disease (LOAD) [1,2,3], but emerging evidence suggests *APOE* plays a role in other neurological conditions, including PD, Parkinson’s related dementia (PDD), and acquired brain injury, defining different clinical subtypes [4,5].

The objective of this review is to present the current evidence on *APOE* as a non-protagonist actor not just in the pathogenesis of major neurodegenerative diseases, such AD and dementia, but in the outcome and stratification of patients with PD, HD, acquired brain injury, coma, and DoC [6,7]. Thus, the discussion on the role of *APOE* alleles in NDs will correlate physiological, clinical and biological information in a novel manner, also integrating the involvement in inflammation processes. These novel features extend the discussion on *APOE* beyond its established role in NDs. Ethical implications of genetic screening for *APOE* variants for disease prevention, and proper information disclosure, will also be briefly discussed.

## 2. Apolipoprotein E: Gene and Protein Structures, Roles and Pathophysiology

### 2.1. Gene Structure and Polymorphisms

**Figure 1:**
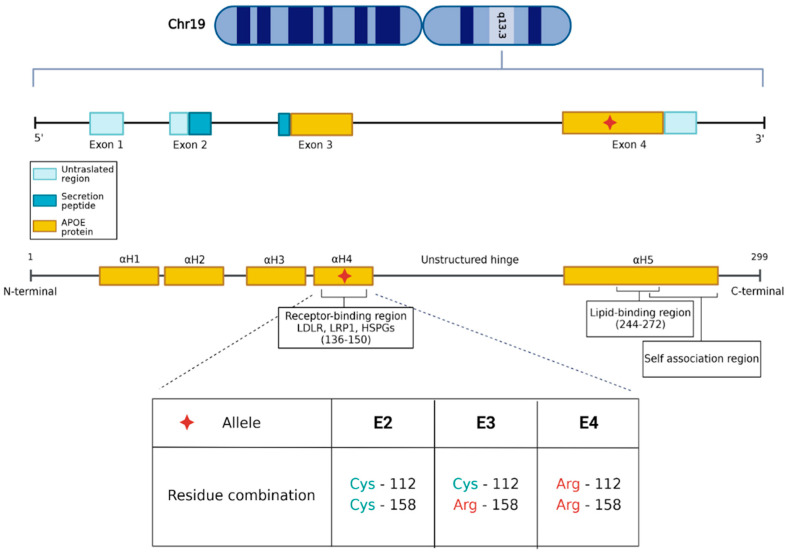
Schematic representation of the *APOE* gene. *APOE* is activated after the cleavage of the secretion peptide and consists of an N-terminal domain comprising four bundled α-helices and a helical C-terminal domain separated by an unstructured hinge region. The N-terminal domain contains the receptor-binding site (residues 136-150), and the C-terminal domain contains the lipid-binding region (residues 244-272) and a self-association region. This image was created with BioRender.com (https://www.biorender.com/, accessed on 15 September 2024).

The apolipoprotein E (*APOE* = gene, ApoE = protein) gene is located on chromosome 19 (q13.3) and is composed of four exons [8]. The gene is located on chromosome 19 (q13.3) and is composed of four exons [8]. *APOE* shows a genetic polymorphism determined by three common alleles, named epsilon2 (E2), epsilon 3 (E3) and 4 (E4), each having differential influences on functional protein and affecting development of different diseases and clinical outcomes (Figure 1). 

The ApoE isoforms are polymorphic on the 112 and 158 residues, with ApoE2 presenting a cysteine on both residues, ApoE3 a cysteine at position 112 and an arginine at position 158, and ApoE4 two arginine residues at both positions [9]. *APOE3* has the highest population incidence, with 50% to 90% frequency in the adult population. Conversely, the other two isoforms are rarer: *APOE4* is present at a frequency of 5 to 35%, and *APOE2* has a frequency of 1–5%. A fourth *APOE* allele is known to exist but is extremely rare, only being observed in three Italian families [10]. Starting from the three common alleles, the most frequent genotypes found in the human population are the homozygous *APOE2/2*, *APOE3/3*, and *APOE4/4*. Heterozygous genotypes that are more common are *APOE2/3*, *APOE2/4*, and *APOE3/4.*

*APOE2* is generally considered a protective factor for AD, having beneficial effects and contrasting disease progression; *APOE3* is neutral, and *APOE4* is detrimental, with a dose-dependent effect. In fact, individuals carrying one *APOE4* allele are at a threefold increased risk, whereas those with two copies face a risk increase of between 9 to 15 times compared to those without any *APOE4* alleles [11]. *APOE4* reportedly has a significant role in worsening of Tau pathology, neuroinflammation and neurodegeneration. In contrast, the *APOE2* allele may act protectively, reducing the risk of LOAD through pathways both dependent and independent of amyloid-beta (Aβ) mechanisms [12].

While being monomorphic in other primates and animals, human *APOE* is the only one to present other functional polymorphisms. In greater apes, the only present form has a partial similarity to the *APOE*4 gene. It is still unknown why only humans have a polymorphic *APOE* gene, although an interesting theory has been proposed, linking the emergence of the alleles to the consumption of meat. During hominid evolution, the spread of meat enriched diets and thus the necessity to metabolize fat through lipoproteins, paired with an extended life span, gave rise to the selection of “meat-adaptive” and “longevity” alleles. Selection of the new isoforms could, therefore, be a result of adaptation to cholesterol and fat metabolism in the new diet of early humans [13]. This theory is corroborated by the diet-associated distribution of *APOE* isoforms across different human populations of the world. Indeed, *APOE3* is more frequent in populations of the Mediterranean basin, where an agricultural economy is long-established, as opposed to populations adapted to inconsistent food availability periods (i.e., Pygmies, aborigines of Malaysia and Australia, Papuans, Native Americans, and Lapps) [14].

However, the most intriguing aspect is the relationship of ApoE with β amyloid (Aβ) protein. Aβ plaques have been observed in aging new world and old world monkeys, suggesting AD-like neuropathy in these species [15,16,17]. The interesting difference in comparison with humans is the greater delay of AD onset in humans after the reproductive period. Since *APOE2* and *APOE3* were selected positively in humans, some evidence suggests that these variants could be beneficial in a species with a growing lifespan with the need to maintain an aging nervous system. Indeed, kin selection and grandmothering could explain the diffusion of the new *APOE* variants as an adaptation of individuals who lived longer and could increase the fitness of their kin and offspring [15,18]. Furthermore, brain enlargement in humans could also have been a driving evolutionary factor for the selection of the E2 and E3 isoforms.

### 2.2. Tissue-Specific Expression and Regulation

Translation of *APOE* occurs at many sites, including the liver and various mononuclear cells. In the brain, it is mainly produced by astrocytes, and more modestly by microglia cells and, in some cases, by neurons themselves [19]. Upstream regulation regions of the genes hold several binding sites for transcription factors [20,21], although the contribution by enhancers is necessary to obtain expression of *APOE*. The nature of the transcription factors and enhancer sequences is often cell and tissue-specific. In astrocytes, ME.1 and ME.2 enhancers promote *APOE* expression. Regulation of *APOE* expression does not seem to be directly associated with AD, with many studies producing discordant results. A study on AD post-mortem brain tissues highlighted a differential transcription of circular and full length APOE RNAs in the frontal lobe compared to the cerebellum, which was used as an internal control [22]. *APOE* also undergoes RNA splicing, producing an incomplete *APOE-I3* that, in the case of brain injury, may be translated into the functioning protein [23].

More information is available on epigenetic regulation of *APOE*. A CpG island is present that overlaps with the 3′-exon (Figure 1), but its influence on gene expression is yet to be unveiled. The 5′-exon includes a CpG island that contains the isoform defining single nucleotide polymorphisms [24]. The C/G ratio of this island is actually altered based on the isoform, with E4 contributing to and E2 disrupting the CpG profile. This feature has been suggested to alter methylation and therefore epigenetic regulation of the *APOE* gene [25].

### 2.3. Protein Structure and Mechanism of Action

**Figure 2:**
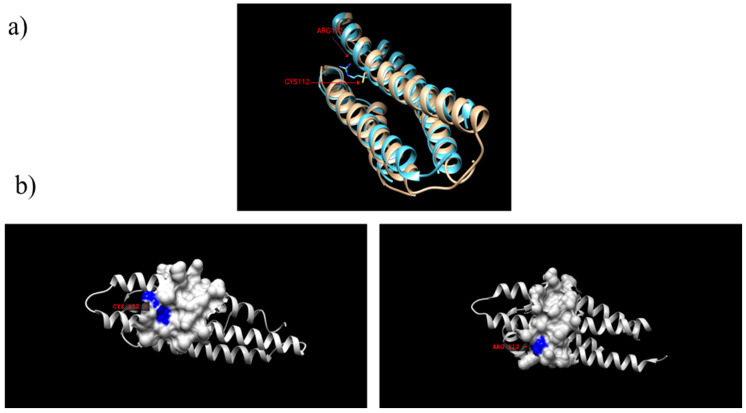
(**a**) 3D model of ApoE3 [24] and ApoE4 [25] α-helix 1 to 4. Superimposed structures of ApoE3 (1BZ4) (blue) and ApoE4 (8AX8) (gray) templates from PDB (RCSB.org) and Uniprot (https://www.uniprot.org/, accessed on 15 September 2024) respectively. The amino acid sequence alignment highlighting C112-R158 polymorphism is also shown. Missense mutations at C112-R158 result in greater exposure of the side chain given the aliphatic nature of the arginine residue. (**b**) Depiction of protein surface differences between ApoE3 and ApoE4. ApoE may exhibit variant-dependent activity in lipid trafficking and Aβ binding. For instance, during coordination with LDLR for lipidation, surface charge is determinant within *in silico* models [28]. Molecular graphics and analyses were performed with UCSF Chimera, developed by the Resource for Biocomputing, Visualization, and Informatics at the University of California, San Francisco, with support from NIH P41-GM103311.

ApoE is a glycoprotein involved in the biological process of lipid transportation and metabolism, contributing to lipid homeostasis [26]. ApoE is an amphipathic protein of 299 amino acids, having approximately a 34 kDa of molecular weight; an unstructured linear hinge connects the N-terminal and C-terminal domains. The mature ApoE protein presents various domains (Low Density Lipid Receptor, LDLR, LDLR Related Protein1, LRP1, and Heparan Sulfate Proteoglycans, HSPG) that interact with distal regions of the protein and with different receptors in the lipid transport metabolism (Figure 1). Amino acid residues at positions 112 and 158 are the main hot-points for allele genetic variations. The C-terminal domain has a better defined tertiary structure with a globular nature that mainly interacts with intracellular components. Conversely, the N-terminal domain has a structure made up of a bundle of four α-helices, one of which contains the low density lipids receptor domain, tasked with interacting with membrane ATP-binding cassette (ABC) transporter 1 [27] during lipid transport. H-bond interactions between the helices deliver a tertiary structure in which the charged residues of the LDLR domain, a membrane receptor of ApoE, are shielded while the neutral side chains are exposed to interact with the target lipids (Figure 2).

**Figure 3:**
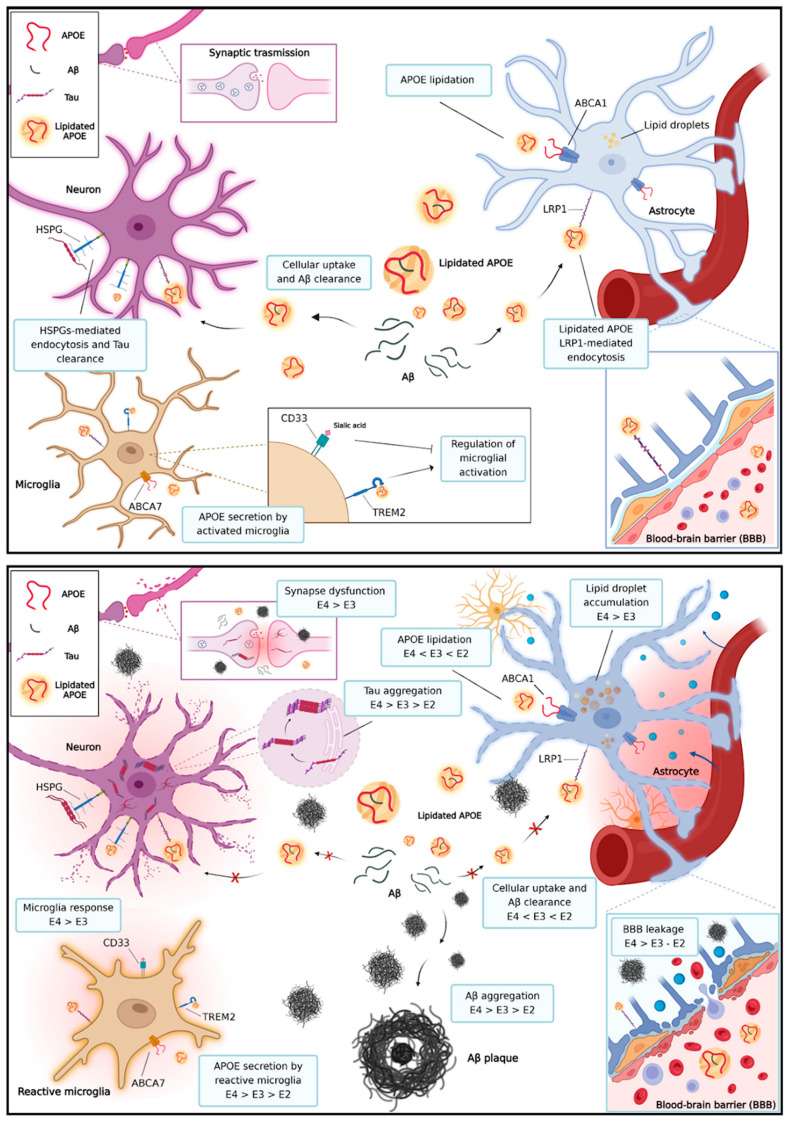
Physiological (top) and pathological (bottom) roles of ApoE in neurodegenerative diseases. ApoE regulates the immunomodulatory activity of microglia and blood–brain barrier integrity. In pathological conditions, ApoE4 promotes the accumulation of lipid droplets in the astrocyte cytoplasm, reduces Aβ uptake, leading to Aβ oligomerization, fibrillation, and seeding into Aβ plaques [1,11,12]. ApoE4 seems to induce hyperphosphorylation of Tau, which accumulates in neurons, leading to the formation of neurofibrillary tangles and synaptic dysfunction [54]. This image was created with BioRender.com (https://www.biorender.com/, accessed on 15 September 2024).

When bound to the cargo lipid, ApoE changes conformation, exposing the hydrophilic residues of the N-terminal, while unfolding the bundle of helices. This conformation is the most commonly found as ApoE is degraded as soon as it is free of its cargo [18,29]. From a functional standpoint, the variation of the amino acid sequence in the low-density lipoprotein receptor domain may alter the ability of the isoforms to fold, interact with receptors, and bind lipids, thus gaining or diminishing αβ-amyloid and Tau balance and homeostasis. Differences in side chain dimensions between C^112^ and R^112^ result in steric differences and consequent differentiation of affinity for lipid species (Figure 2). The R112 of ApoE4 is known to influence protein structure and flexibility [30]. Indeed, R112 may interact with spatially adjacent residues 5–21 and 271–279, altering non covalent bonding between side chains of these domains. The result of this new conformation has been proposed to be an increased flexibility of motion of ApoE4 compared to ApoE3, possibly explaining the ability of the E4 variant to bind VLDL [30]. The structural properties of ApoE isoforms are critical in determining the protein’s interaction with cellular receptors and lipids, thereby impacting processes such as lipid transport, neuronal repair, and immune modulation within the brain. Under physiological conditions, ApoE is loaded with lipids and transported by the Abca1 lipid transposase protein. Once localized in the interstitial fluid outside of neurons and glia cells, ApoE carries lipids and HDL-like particles through the brain blood barriers (BBB) (Figure 3). The singular isoforms differ in various ways, such as the quality of lipids and proteoglycans, the dimensions of the lipid particles that ApoE is able to bind, and receptor activity. C112 and R158 are both positioned in determining sites for lipid and receptor interaction. Proof of this can be found outside of the context of the brain, i.e., in peripheral tissues; ApoE3 and ApoE2 have a substantial affinity for HDL, whereas ApoE4 binds preferentially to VLDL [31]. In ApoE4 carriers, the increased affinity of the variant for plasma cholesterol correlates with a downregulated expression of LDR through cell negative feedback, thus raising plasma cholesterol levels [32]. Thus, the presented polymorphic differences may have a central role in determining the efficiency of lipid trafficking in the brain, contributing to neurodegeneration and inflammation pathologies. Furthermore, C158 in ApoE2 is located within the receptor binding domain, resulting in an alteration of charge and thus binding affinity [33]. Missense mutations at C112-R158 have not yet been linked directly to pathophysiological mechanisms that lead to neurodegeneration diseases, but their prevalence is known in other pathological conditions related to lipid trafficking, such as type III hyperlipoproteinemia (HLP). All of these characteristics will therefore be determining in brain physiology and disease development [34]. Understanding these biochemical dynamics is essential for elucidating ApoE’s involvement in many neurological processes and diseases.

### 2.4. ApoE Functions

The main recognized role of ApoE is to bind lipids, mainly low density lipids and cholesterol secreted by cells of the glia, and direct them towards neuron growth and repair in case of damage. In particular, the lipids are shuttled by ApoE to neurons to improve synaptic plasticity, synaptogenesis, and axonal regeneration [35,36] (Figure 3). In addition, ApoE is involved in vascular integrity, glucose metabolism, and mitochondrial function [37,38,39]. ApoE is lipidated by the lipid transporter ABCA1, located in the astrocyte plasma membrane, and ABCA7, located in the microglia plasma membrane. Lipidated ApoE interacts with the amyloid-β peptide (Aβ) and promotes cellular uptake and clearance. Lipidated ApoE is internalized by astrocytes, microglia, neurons, and endothelial cells via LRP1-mediated endocytosis. The mechanism is involved in cellular uptake and Aβ clearance. ApoE interacts with TREM2 in the microglia plasma membrane and promotes microglial activation.

Further, an emerging role is also being hypothesized in the endosomal–lysosomal system, in particular related to neuron development and maintenance. Abnormalities in endosomal trafficking and lysosome maturation have implications for many components, including ApoE, thus contributing to the pathophysiology of neurodegeneration. LDLR related receptor (LDR) is a membrane receptor of ApoE that directly interacts with the LDLR domain. In particular, the LDR–ApoE axis of lipid transport seems to be a determinant in AD development and deposition of Aβ aggregates [40,41].

### 2.5. ApoE and Neuroinflammation

In many circumstances, including CNS injury, the balance between inflammatory and intrinsic repair processes regulates functional recovery; however, long periods of continuous inflammation caused by disease-related symptoms (i.e., Aβ deposition, Tau phosphorylation) and/or environmental factors (i.e., trauma, stroke, chronic infection, diet) are known to induce synaptic dysfunction and enhance the risk of developing late and secondary neurodegeneration (Figure 3) [42,43]. Recently, the involvement of APOE in persistence of chronic inflammation and the development of neurodegeneration has emerged. In patients with TBI, a long-lasting neuroinflammatory cascade may lead to the progression of brain damage, favoring neurodegeneration and cognitive impairment. Indeed, increased levels of a proinflammatory cytokine, interleukin (IL)-18, correlate with the patients’ cognitive impairment and disability severity in chronic TBI patients [44].

A study hypothesized *APOE* participating in progressive differentiation of disease associated microglia, which starts from a homeostatic phase that advances into an intermediate phase in which both *APOE* and lipid metabolism genes are partially activated [45]. Rather than acting as “bystanders” in AD development, brain immune cells develop a typical disease related expression profile which mediates the immune response. Specific subpopulations of microglia cells (i.e., disease associated microglia, DAM) in AD murine models have been sorted, and unique profiles of immune response and phagocytosis of aggregates have been observed. Reportedly, biomarkers purinergic receptor *P2Y12* and *P2Y13* (*P2ry12/P2ry13*), CX3C motif chemokine receptor 1 (*CX3CR1*), transmembrane protein 119 (*TMEM119*), and (Triggering Receptor Expressed On Myeloid Cells 2) (*TREM2*) were deregulated, along with *APOE* [45]. Knockout experiments of TREM2 determined that the mechanism is part of a temporarily coupled pathway required to differentiate DAM cells. It is important in this context to highlight the upregulation of lipid metabolic genes, including, most importantly, *APOE*. This indicates the heightened activity of neuron maintenance by the resident immune cells, although this process is observed in later stages of the disease [45]. Extracellular Tau binds heparan sulfate proteoglycans (HSPGs) on the neuron surface. HSPG-mediated endocytosis is involved in cellular uptake and aggregated Tau clearance.

In TREM2-silenced mouse models, transcriptome analysis showed modest differences in deregulated gene populations between *TREM2ko/APOE3* and *TREM2ko/APOE4* compared to the relative Trem2 positive subjects. This feature suggests a key role of TREM2 in eliciting the immune response, compared to the role of the variants. More precisely, TREM2 silencing “blunts” the ApoE variant contribution to the immune response against plaque deposition [46]. In further studies, ApoE has been demonstrated to actively promote the phenotypic switch of glia from the homeostatic to the activated state, in collaboration with Trem2. In murine models, age and *APOE4* burden correlated positively with terminal glial exhaustion and heightened expression of inflammatory NF-kB, IRF family, and interferon correlated regulons. In AD patients, similar conditions were found, with exhausted microglial being more abundant in affected patients compared to healthy subjects. Interestingly, *APOE4* carriers with AD showed enriched populations of terminally inflamed glial cells [47].

*APOE* also regulates activation and differentiation of glial cells and the modulation of neuroinflammation, through the ApoE–Trem2 axis (Figure 3), but the contribution of the different *APOE* isoforms remains an open matter still to be defined. In neuroinflammation, the activation of resident glial cells, microglia, astrocytes, endothelial cells, and peripherally derived immune cells, leads to the diffuse production of several pro-inflammatory cytokines [48].

In murine models, age and *APOE4* burden correlated positively with terminal glial exhaustion and heightened expression of inflammatory NF-kB, interferon correlated regulons, and TGF-β regulated checkpoints [19,47,49]. In AD patients, similar conditions were found, with exhausted microglial being more abundant in affected patients compared to healthy subjects. Interestingly, *APOE4* carriers with AD showed enriched populations of terminally inflamed glial cells [47]. 

Indeed, Trem2 and ApoE interaction represents a pivotal point in Aβ clearance since microglia have been observed to be more effective in Aβ phagocytosis when lipidated ApoE is bound to Trem2 [50]. Further evidence of this relation can be found in further studies on *APOE4* carriers from the ADNI database, where cognitive decline, Tau phosphorylation, Aβ deposition, Trem2 baseline values and neurodegeneration (i.e., hippocampal volume change rates) have been investigated. Results showed a trend in which increased Trem2 levels are correlated with subdued *APOE4* associated symptoms and attenuated risk for AD neurodegeneration [51]. In contrast, experiments with animal models revealed an amelioration of Tau pathology after *TREM2* deletion [52]. Conversely, the microglial response is inhibited by CD33, a receptor that binds sialoproteins, such as sialic acid [52]. Future research on the therapeutic influence of Trem2 could benefit from an investigation of the roles of *APOE* alleles.

In the brain tissue of human subjects with amyotrophic lateral sclerosis, multiple sclerosis (MS), and AD, an *APOE* molecular signature was associated with activated phagocytic phenotype glia near the amyloid plaques [53]. In a presented molecular model, glia cells are kept in M0 phase through TGFβ regulation of homeostatic genes, namely *P2RY12, TMEM119*, G protein-coupled receptor 34 (*GPR3*4), Jun proto-oncogene (*JUN*), Olfactomedin Like 3 (*OLFML3*), Colony stimulating factor 1 receptor (*CSFLR*), β-hexosaminidase subunit β (HEXB), Proto-oncogene tyrosine-protein kinase MER (*MERTK*), Ras homolog gene family member B *RHOB, CX3CRL*, Transforming growth factor β receptor I (*TGFBR1*), and Transforming Growth Factor β 1 (*TGFB1*), Myocyte-Specific Enhancer Factor 2A (*MEF2A)*, V-Maf Musculoaponeurotic Fibrosarcoma Oncogene Homolog B (*MAFB*), Sal-like 1 (*SALL1*), and Early growth response protein 1 (*EGR1*) transcription factors. When the Trem2 membrane receptor is activated by a neuritic amyloid plaque, two parallel cascades suppress TGFβ regulation and promote *APOE* to activate a microglial neurodegenerative phenotype. In this subset of glial cells, *APOE* seems modulate transcriptional and post-transcriptional upregulation of several inflammatory genes (i.e., C-type lectin domain family 7 member A (*CLEC7A*), Galectin-3 (*LGALS3*), Integrin Subunit α X (*ITGAX*) and C-X-C motif chemokine ligand 10 (*CXCL10*). Interestingly, this represents an interesting field of investigation, especially for clarifying the role of Apoe as a modulator of inflammatory response, and above all as a possible therapeutic target.

## 3. APOE and Neurodegenerative Diseases: A Non-Protagonist Role

In the following sections, we will discuss the pathophysiology of major neurodegenerative diseases, AD, and PD, from the perspective of *APOE*’s non-protagonist role in pathophysiological processes (i.e., amyloid homeostasis, Tau phosphorylation, synuclein pathologies).

### 3.1. ApoE and Alzheimer’s Disease

AD is a chronic progressive neurodegenerative disease, responsible for 60 to 70% of dementia cases [55,56]. Early Onset AD (EOAD) refers to familial forms associated with one of the three known autosomal dominant mutations, Amyloid Precursor Protein (APP), Presenilin 1 (PSEN1), or Presenilin 2 (PSEN2), and comprises about 5% to 6% of cases, with subjects developing symptoms before reaching 65 years of age. Sporadic Late Onset AD, which represents almost 80% of AD cases [55], is a multifactorial condition that often manifests after 65–70 years of age with mild symptoms in different cognitive domains, e.g., memory, behavior, emotional instability, disorientation, aphasia, and eventually loss of organ function. In these late sporadic forms, inheritance of the *APOE4* allele is considered the strongest genetic risk factor [2,57,58]. From a clinical point of view, the *APO*E genotype has been shown to affect the age of onset of AD (APOE*4* < *APOE3* < A*POE2)*, demonstrating its central role in the pathogenesis and progression of the disease. Advanced omics research techniques, including single-cell analyses, transcriptomics, and genome-wide association studies (GWAS), have provided interesting insights into how *APOE* interacts with various biological pathways to influence Alzheimer’s pathology. Such studies have revealed over 44 risk loci associated with LOAD, enhancing our understanding of the genetic landscape of the disease [54,59]. GWAS studies have revealed substantial evidence of risk loci for neurodegeneration, and, in recent times, an investigation identified 11 loci highly associated with AD, PD, dementias, and amyotrophic lateral sclerosis [53]. Nevertheless, it is important to note the limitations of these types of studies, e.g., the authors claim that an overly conservative threshold for shared loci may have hindered the associated loci detection. Further limitations that are common among GWAS studies are the assignment of random genes to examine for association [60]. Data redundancy in databases should also be taken into account for the success of GWAS studies, as diagnostic information can often differ from the actual pathological status of the subjects.

Neuropathological hallmarks of AD are deposition of extracellular amyloid Aβ plaques and abnormally phosphorylated Tau that accumulates intraneuronally to form neurofibrillary tangles (NFTs) in cortex and hippocampus, leading to progressive neuronal death and synaptic dysfunction (Figure 3). Glial cells and astrocytes, the major immune cells tasked with protecting neurons, become gradually deregulated, favoring neuroinflammation and oxidative stress [61]. Changes in levels of specific proteins in cerebrospinal fluid (CSF) and in blood, e.g., Aβ42 and Aβ0, total and phosphorylated Tau (pTau), reflect pathological processes occurring in brain tissue, supporting clinical diagnostic procedures [62,63]. The ApoE–Aβ interaction regulates Aβ aggregation and clearance and therefore directly influences the development of amyloid plaques and subsequent Tau related pathology [58]. In particular, ApoE affects the pathological aggregation of Aβ, with ApoE4 promoting the formation of Aβ oligomers and fibrils, the most neurotoxic species in AD, in comparison to ApoE2 or ApoE3 [64,65,66,67,68]. Concurrently, when AD has reached a significant point of progression glial cells and astrocytes can be observed in a heightened state of activity in the attempt to phagocytize the plaques [61]. The hypothesis of *APOE4* directly modulating this reaction has been put forward, with some evidence pointing towards an increase in stress related markers.

During AD development, isoform-dependent decreased activity of ApoE has been proved to hinder lipid trafficking and Aβ clearance, resulting in a pathological microenvironment (Figure 3). In vitro inhibition of LDLR related receptor activity has been found to increase Aβ deposition [40,41], mainly due to the influence of ApoE4 on LDR activity and expression. In healthy subjects, LDR physiologically decreases with age, but this pattern is heightened in *APOE4* carriers. Furthermore, higher levels of LDR effectively ameliorate Aβ clearance, but only in *APOE3* and *APOE2,* implying an isoform-dependent interaction. In the case of *APOE4* variants, the increased LDR profile is not enough to revert the detrimental effect of this isoform. *APOE4* also has an influence on endosome production and dimension, with carriers exhibiting enlargement of early endosomes. Indeed, endosome volume in neurons was compared between carriers of the three alleles, highlighting an increase in the radius of the endosomes in E4 variant carriers. Since the observed modulation of endosome dimensions begins early, before the onset of AD, targeting of this feature has been proposed as a therapeutic strategy to facilitate Aβ clearance [69].

Many biomarker studies have assessed the impact of *APOE* on the changes in circulating levels, but results are conflicting. *APOE4* carriers show lower levels of CSF Aβ, but no differences in Tau [70]; by integrating ATN classification for AD, a dose-dependent effect has been suggested, with ApoE phenotypes at higher risk of developing AD being associated with a lower CSF Aβ42/40 ratio [71]. Recently, a large multicenter study of 500 *APOE4* homozygotes revealed that these subjects exhibit the main characteristics of genetically determined AD dementia, namely near-full penetrance, symptom onset predictability and a predictable sequence of biomarker and clinical changes [70].

Despite significant advancements, many genetic factors remain uncharacterized, highlighting the need for continued research to fully unravel the multifactorial roles of *APOE* and other genetic components in AD.

### 3.2. ApoE and Parkinson’s Disease

Parkinson’s disease (PD) is a progressive degenerative disease mainly characterized by the steady loss of movement control and a host of symptoms depending on the severity. Early stage symptoms of PD include tremor of the hands, rigidity in movement and bradykinesia and are sometimes limited to a single side of the body. In more advanced cases, PD manifests with non-motor related symptoms such as sleep disturbances, behavioral changes, psychosis and late development of Parkinson’s disease dementia (PDD) [72,73]. The cause of PD is yet to be uncovered. Anatomopathological hallmarks of PD are consistent with death of dopamine producing neurons and loss of melanized neurons in the substantia nigra [74], crucial for movement coordination [75], with formation of Lewy bodies [74] composed of misfolded α-synuclein protein (ASyn). In particular, ASyn neuropathy elicits an immune response, by means of glial activation, through mitochondrial oxidative stress [76].

A number of mutations has been positively linked to rare familiar forms of the disease, including Leucine-rich repeat kinase 2 (*LRRK2*), Synuclein α (*SNCA*) and Vacuolar protein sorting ortholog 35 (*VPS35*), and PTEN-induced kinase 1 (*PINK1*), Parkin *(PRKN*) and Parkinson disease protein 7 (*DJ1*) [73]. Further, other environmental factors, including exposure to pesticides (glyphosate, paraquat, benomyl) and heavy metals (iron, manganese, lead, mercury) [77] as well as polymorphisms in SNCA gene, which codes for the α-synuclein protein (ASyn), a key protein in PD pathology, have been suggested to increase the risk of developing the disease.

Several studies on *APOE* and PD have found an association between *APOE2*, *APOE3* and *APOE4* variants and disease progression and susceptibility, although clinical and neurochemical correlates have not been completely established. Moreover, *APOE* variants play a differential role in determining α-synucleinopathies, e.g., PD, Lewy bodies dementia, and multiple system atrophy (MSA) [74]. With more caution compared to AD, *APOE* and its isoforms are considered to be risk factors for PD development, thus representing a marker for patients stratification due to their association with characteristic clinical phenotypes or biochemical patterns. This uncertainty is due to conflicting results reported in the scientific literature concerning the *APOE* contribution to PD, mainly due to patient selection, limited cohort, and lack of standardization in scales and methods. In fact, in some cases, *APOE2* is considered beneficial [78] while in others, it is associated with a high risk of the sporadic form of the disease [79]. As a therapeutic approach target, *APOE* could represent a viable resource in ameliorating PD neurodegeneration. Indeed, in SN tissues of subjects presenting LBs, ApoE’s main receptor LRP1 was found to be overexpressed, indicating a possible underlying mechanism of PD linked to lipid transport, signaling homeostasis and α-synuclein deposition [74]. A study on 175 PD patients and 89 non-neurodegenerative controls, categorized by *APOE4* carrier status, showed that *APOE4* PD subjects have significantly lower levels of Aβ42 in CSF compared to both PD patients without *APOE*4 and the control group, independently of age. Additionally, PD patients with *APOE4* are clinically characterized by higher non-motor symptoms [80].

Furthermore, studies support the contribution of ApoE4 in developing dementia among PD patients, probably by participating in the formation of toxic aggregated Aβ42 species, or due to the co-occurrence of AD pathology. A meta-analysis of 17 studies, including a total of 820 PDD patients and 1922 non-PDD subjects, demonstrated that *APOE4* is associated with a higher risk of developing PDD [72], with *APOE2* having either a risk or a protective role. Moreover, *APOE4* was linked to a steeper cognitive decline in PDD human subjects compared to healthy subjects [81], although this result was found to be in contrast with further studies [82].

Within PD cases, a striking contribution to PDD progression was also linked to the interaction between *APOE* variants and alleles of its receptor *LRP*, specifically *LRP1B*. In a genome-wide survival meta-analysis of 3923 clinically diagnosed European PD cases, *LRP1B* carriers showed a heightened hazard of progression to PDD regardless of *APOE4* presence. Nevertheless, double positive *APOE4* and *LRP1B* subjects showed the highest scores in PDD progression hazard [83]. *LRP1B* is a type of LDL receptor that is expressed in many tissues, including the brain. Apart from LDL, *LRP1B* is involved in Aβ [84] and Tau [85] trafficking activity.

In the future, the application of high throughput technologies to large collections of biological samples, from well-characterized cohorts, will shed more light on this complex field [43].

### 3.3. ApoE and Disorders of Consciousness

Disorders of consciousness are states characterized by alterations in awareness, often caused by severe acquired brain injury (sABI), mainly of traumatic or vascular origin. DoC encompass several clinical states, including coma, unresponsive wakefulness syndrome (UWS), also known as vegetative state (VS), and minimally conscious state (MCS), which are defined as “prolonged” when a DoC lasts more than 28 days. These conditions are often transient but can also exist as a permanent outcome.

The continuing evolution of knowledge within the spectrum of DoC arises from the growing need to clarify the neuropathological overlap between UWS and other clinical conditions, such as MCS and locked-in syndrome (LIS) and to identify predictive molecular or biological markers for patient stratification [86,87,88].

About 30 years ago, the first studies began to suggest an association between the possession of the *APOE4* allele and an increase in adverse outcomes following TBI. In fact, patients carrying the *APOE4* allele showed increased brain Aβ deposition, and a higher frequency of fatal outcomes [89,90]. Moreover, after brain injury, the presence or absence of the *APOE4* allele has been revealed to be a better predictor of outcome than age, duration of coma, or Glasgow Coma Scale (GSC) assessed at admission [91]. Interestingly, patients who did not regain consciousness had a higher frequency of the *APOE4* allele, and an absence of the *APOE2* allele [91], suggesting a possible role for *APOE* genotypes in determining genetic susceptibility to the effects of TBI. 

Furthermore, among 44 patients with intracerebral hemorrhage, those with the *APOE4* allele had a worse neurologic outcome (lower Barthel score) than patients without the E4 allele, and an approximately three-fold increase in mortality [92]. Subsequent studies also confirmed differences in clinical outcomes based on *APOE* genotype, with *APOE4* carriers having reduced recovery potential during rehabilitation after TBI [93,94,95,96]. Oppositely, there was little or no evidence of an association between *APOE* genotype and outcome in mild or moderate injuries [97,98].

In a cohort of 648 TBI patients with documented loss of consciousness or posttraumatic amnesia, the *APOE4* allele was associated with worse long-term outcome over one to five years, but not with acute injury severity as measured by GCS [99]. On the Glasgow Outcome Scale-Extended (GOSE), a greater proportion of *APOE4* individuals (26.61%) had severe disability compared with non *APOE4* carriers (*n* = 453, *p* = 0.01) with greater susceptibility observed in females [99].

In agreement, a meta-analysis of a total of 736 *APOE4* positive and 1791 *APOE4* negative patients who had sustained severe to mild head trauma demonstrated that the presence of the *APOE4* allele is not associated with the initial severity of brain damage following head trauma, but is associated with an increased risk of poor long-term outcome at six months after injury [100].

A large study of 984 patients with TBI showed conflicting results, with no difference between *APOE* genotypes and outcome, with unfavorable outcome observed in 118/324 (36%) of *APOE4* carriers compared with 215/660 (33%) of *APOE4* non carriers [90]. Notwithstanding, in this large cohort, possession of *APOE4* reduced the prediction of a favorable outcome in children and young adults, suggesting that *APOE* may be involved in the repair and recovery processes after TBI [101].

Evidence from experimental and clinical studies of brain injury suggests that *APOE* acts as a player in the brain’s response to injury. However, disagreement between studies may again be related to sample size and associated power limitations, as well as patient selection, which differ in terms of injury severity, observation period, and outcome assessment method. Although the precise role and relevance of *APOE* in severe TBI and DoC remain to be confirmed, the apparently detrimental effects of the E4 allele may be related to one or more proposed functions of this lipoprotein. In particular, the detrimental effect of E4 on TBI outcome may also be associated with the binding and deposition of Aβ, which may potentiate an immune response, preventing recovery for E4 carriers [96].

### 3.4. ApoE as a Target in Therapeutic Strategies

Currently, no drugs for AD are available and unique FDA (Food and Drug Administration, USA)-approved therapies aim at either mitigating symptoms or slowing disease progression. Therapies for NDs are continuously searching for targets and ApoE represents a viable target, both in itself and in the physiological processes in which it is involved in (i.e., amyloid metabolism). Current therapies targeting ApoE are diversified based on the approach which may focus on regulation of expression, lipidation, immune-modulation and Aβ subtraction. In general, developing therapeutic strategies aim to reduce amyloid plaque burden and act as prophylactic measures to lower ND development, but collateral effects may occur.

Therapies that exploit the beneficial effects of *APOE2* and *APOE3* can regulate the expression of these alleles to mitigate the detrimental effects of *APOE4* in heterozygous subjects. In this regard, in vivo gene therapy on murine models has yielded interesting results, with significant shrinking of Aβ plaques in AD models following adenovirus-associated secretion of *APOE2* [102]. Concurrently, modulation markers of neuroinflammation and activation of neural microglial showed a systemic improvement after *APOE2* induction [102]. RNA interference (RNAi) also represents a viable option for the modulation of allele expression. Recently, small interfering RNAs have been successfully produced and utilized for RNAi mediated loss of function of ApoE4 in mice, with promising results on immune modulation. Success of the RNAi was attributed, among other factors, to the efficient internalization of the molecular scaffold responsible for divalent siRNA transcription [103]. Genome-editing through CRISPR/Cas9 protocols, which rely on the advantageous ability to insert point mutations, has been developed to act directly on *APOE4.* Indeed, by exploiting the SNP of the *APOE* gene, it becomes advantageous to implement CRISPR/Cas9 in order to revert the detrimental phenotype [104].

Immunotherapy-based strategies have also been studied. One study investigated the effects on amyloid deposition in mice after administration of anti-ApoE antibodies. The objective of the study was to facilitate microglial-assisted amyloid clearance, similar to the effect induced by anti-amyloid antibodies. Short-term results showed activation of microglia, and in the long-term, Aβ40 and Aβ42 levels significantly decreased [105].

An alternative to targeting ApoE is to develop strategies that modulate the availability of ApoE substrates. Namely, strategies that regulate the concentration of ApoE substrates are anti-Aβ immunotherapy and enhancement of ApoE lipidation. Anti-Aβ therapies have reached FDA approval with the development and use of aducanumab and lecanemab as anti-Aβ pharmaceuticals. Through administration of aducanumab and lecanemab, the aim is to increase Aβ1–42 clearance and induce a host immune response, in particular, microglial activation [106,107]. Despite the promising results, some complications related to anti-Aβ therapy have arisen. During clinical trials with aducanumab, lecanemab and other drugs, amyloid related imaging abnormalities (ARIA) have been registered with the development of cerebral hemorrhage and brain edema [108]. Interestingly, ApoE4 was linked to a higher risk of developing ARIA during anti-Aβ immunotherapy, highlighting the greater difficulty of carriers in disease treatment. Reportedly, *APOE4* worsens ARIA through continuous inflammation and immune dysregulation, reduced cerebral integrity and elevated cerebral amyloid angiopathy [109].

Enzymatic activity of ApoE is also a target for therapeutic research. It is established that ApoE exhibits a higher rate of Aβ clearance and promotion of brain homeostasis when it is lipidated, therefore experimental therapies have been developed to regulate the equilibrium between lipidated and non-lipidated ApoE. In this regard, the induction of an ABCA1 agonist (bexarotene) raises ABCA1 expression in vitro and thus lipidation of ApoE4 in vivo. Limitations of this strategy are related to the multiple receptors of bexarotene and thus researchers have investigated effective ways to target ABCA1 [110].

## 4. Bioethics and *APOE* Alleles: Information Disclosure and Clinical Path

As presented, current understanding of AD states that while *APOE4* represents the greatest risk for development of the disease [111], the presence of the allele does not determine AD onset *a priori*. In other words, *APOE4* does not represent a solid biomarker for AD diagnosis [112]. Nevertheless, genetic screening for *APOE* carrier status could represent a source of information on the future health of interested subjects, but its use (or abuse) should be considered carefully. In clinical settings, diagnosing neurodegenerative disorders can be hindered by subclinical manifestations of symptoms. In this case, genetic testing for *APOE* variants has been demonstrated to be helpful in determining the pathological status of subjects, and therefore in establishing the proper clinical path [113]. In 1993, “The Statement on use of apolipoprotein E testing for Alzheimer disease” by the American College of Medical Genetics, American Society of Human Genetics Working Group on *APOE* and Alzheimer disease and the American Psychological Association, defined the uses and limitations of *APOE4* screening in the context of AD preventive care. While considering *APOE4* as a clear risk factor for AD development, its use outside of clinical care context has been clearly discouraged. Indeed, genetic information should be disclosed and discussed with healthcare professionals and integrated with the subject’s medical history, in order to clearly articulate the implications that arise [112].

Screening for *APOE4* is not part of the preventive care for AD and dementia and, because of this, carriers are unaware of their genotype. Genetic testing is usually carried out after any cognitive symptoms have already emerged, with the aim of understanding the differential diagnosis, possible clinical outcomes, and programming future care of the affected patient [114,115]. The principal issue of *APOE* genotyping outside of clinical settings is connected to the misinterpretation of genetic information, resulting in potential overreactions and social alienation. 

Alternatively, as a possible result, individuals who undergo testing and discover they are *APOE4* carriers could decide to adopt a proactive lifestyle, changing modifiable risk factors such as smoking, diet, physical inactivity, etc., which account for an increased risk of developing AD [114].

Subjects seeking genetic testing for *APOE* status range from interested healthy subjects, to subjects with family history, to patients diagnosed with AD who already have other clinical results already confirming the onset of AD. Although commercially accessible to non-neurological subjects, eligibility for genetic screening has not yet been clearly defined, and since disclosure of *APOE* allele status to patients and concerned subjects has ethical implications, careful consideration of the psychological and social consequences must be considered [116].

## 5. Concluding Remarks

The incidence rate of neurological disorders has increased in the last few decades, following a demographic associated trend closely related to the aging demography of industrialized countries [117]. There is extensive evidence that *APOE* plays a central role in modulating AD pathogenesis by varying effects on net oligomerization/aggregation of Aβ and Tau, as well as various inflammatory and recovery pathways, and response in clinical trials. However, other mechanisms have yet to be identified, which may determine the involvement of *APOE* in other neurological pathologies, both degenerative and in acquired BI, participating in repair and recovery. Nevertheless, we must not expect the role of ApoE to be universal in NDs, as suggested by observations in HD patients. In fact, in a study on a Chinese cohort of 223 patients, no association was found between *APOE* genotypes and age of onset, motor-onset, and non-motor-onset [118]. Furthermore, in patients with HD, the frequency of the *APOE4* allele was statistically lower than that in controls (7.1 vs. 12.0%) [118]. Similarly, in 145 patients symptomatic for HD with psychiatric and somatic symptoms (depression, psychosis, dementia, choreic, and other movement disorders), no significant effects of the *APOE4* allele were observed regarding clinical characteristics including age of onset, nor were sex differences for the *APOE2/APOE3* genotype observed [119], oppositely to a previous study [120].

The finding that *APOE* does not appear to be a genetic modifier for HD may be partly explained by the fact that HD is an autosomal dominant degenerative disease, which implies different mechanisms with respect to other multifactorial diseases.

The aim of this work was to present the current understanding of *APOE* involvement in the pathophysiology of neurological disorders, highlighting its role as a non-protagonist actor. Furthermore, consideration of the ethical and social implications of *APOE* variant testing and genetic information disclosure were deemed relevant and were therefore discussed. 

*APOE* gene structure and expression were illustrated, presenting the protein domains that have an active role in binding lipids and interacting with receptors of astrocytes and glia cells, i.e., ABCA1 and LRP1. Functions of ApoE in physiological and pathological conditions were presented (Figure 3), particularly focusing on ApoE-mediated Aβ clearance andthe differential ability of the isoforms to enable Aβ aggregate formation. In particular, emphasis was placed on the ability of APOE4 to concurrently mediate Aβ aggregate nucleation and reduce cellular uptake of Aβ oligomers. Furthermore, steric differences caused by the polymorphic variants were depicted using the Chimera 3D modeling software Version 1.14 (build 42094) and templates from PDB and Uniprot databases [121,122], in order to illustrate the functional differences between ApoE3 and ApoE4.

Thus, the role of *APOE* variants in contributing to neuroinflammation was also discussed, highlighting the central role of the molecular cross-talk between APOE and TREM2. As discussed, the *APOE–TREM2* axis determines microglial activation and therefore modulates inflammation, with ApoE4 eliciting a stronger inflammatory response. 

In this review, a focus was given on the differential contribution of the different *APOE* variants to neuropathies and ND clinical outcomes. In comparison with the neutral *APOE3* and protective *APOE2* alleles, *APOE4* remains the most important genetic risk factor for AD, although it is still unclear whether the pathogenic role results from a toxic gain of function or loss of protective function. Further, findings suggest that *APOE* correlates with specific clinical features among PD patients, as well as in recovery potential in severe acquired brain injury. Further studies on the role of genetic factors in the efficacy of neurorehabilitation are also warranted. 

Finally, the authors deemed it necessary to address the ethical issues involved in genetic screening for *APOE* variants. The weight of the disclosed information changes, as subjects seeking such tests range from ND patients to healthy subjects. For these reasons, according to the “Statement on use of apolipoprotein E testing for Alzheimer disease”, the authors argue that genetic testing should be kept within the confines of clinical care and informed consent, in order to minimize the social and psychological damage generated by misunderstood information.
